# Use of Azathioprine in Ulcerative Colitis: A Comprehensive Review

**DOI:** 10.7759/cureus.24874

**Published:** 2022-05-10

**Authors:** Bipadabhanjan Mallick, Sarthak Malik

**Affiliations:** 1 Gastroenterology, Kalinga Institute of Medical Sciences (KIMS), Bhubaneswar, IND; 2 Gastroenterology, Fortis Escorts Heart Institute, New Delhi, IND

**Keywords:** adverse drug reaction, efficacy, ulcerative colitis, inflammatory bowel disease, azathioprine

## Abstract

Ulcerative colitis (UC) is a relapsing and remitting chronic inflammatory disease of the large intestine characterized by bloody diarrhea, abdominal pain, urgency, and tenesmus. Rapid induction and maintenance of remission are the primary goals of treatment. Azathioprine (AZA), a purine analog, has been utilized as an immuno-modulator to maintain remission in UC. AZA has been used for a long time, but there is still controversy about its effectiveness, drug interactions, and side effects in people with UC. We conducted a comprehensive analysis of the literature and present a detailed insight into the role of AZA in patients with UC.

## Introduction and background

Ulcerative colitis (UC) is a chronic inflammatory disorder of the colon characterized by periods of relapse and remission. Remission is negatively associated with the future occurrence of clinical flares, hospitalization, use of steroids, and complications like dysplasia and colectomy. The rapid induction and maintenance of remission are the main principles of treatment for UC. Corticosteroids, 5-aminosalicylates (5-ASA), immunomodulators, biologics, and small molecules are the foundation of UC treatment. The most cost-effective immunomodulators, such as azathioprine (AZA), have been used to treat UC for many decades, primarily to maintain remission [1,2].

## Review

Metabolism

**Figure 1:**
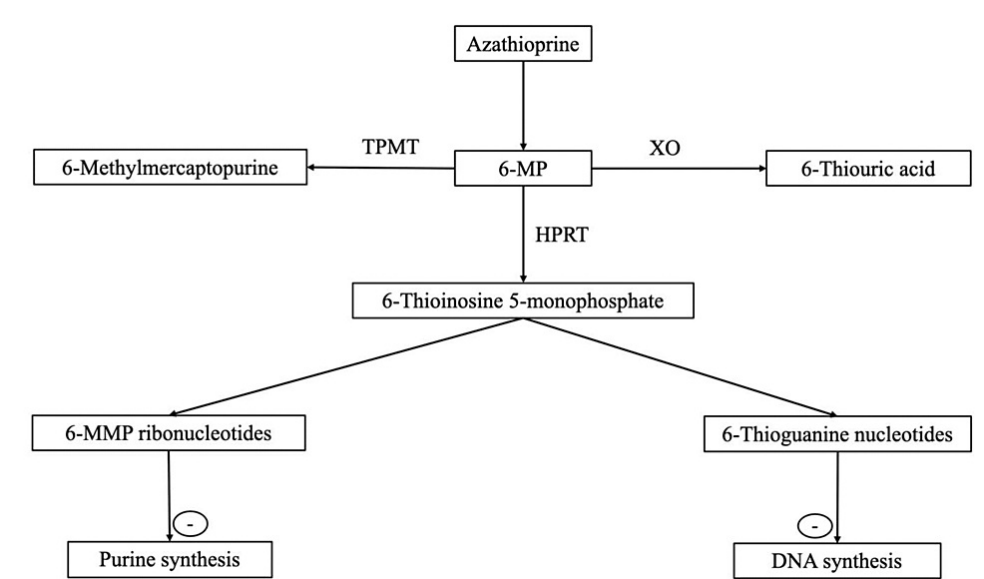
Schematic representation of azathioprine metabolism TPMT: thiopurine-S-methyltransferase; 6-MP: 6-mercaptopurine; XO: xanthine oxidase; 6-MMP: 6-methylmercaptopurine; HPRT: hypoxanthine-guanine phosphoribosyltransferase

Azathioprine is a pro-drug that undergoes a complicated metabolic transformation to become pharmacologically active as 6-thioguanine nucleotides (6-TGN) [3]. AZA is transformed to 6-mercaptopurine (6-MP) either in the absence of enzymes or with the action of the glutathione S-transferase enzyme. Three competing enzymes, xanthine oxidase (XO), thiopurine-S-methyltransferase (TPMT), and hypoxanthine phosphoribosyltransferase, metabolize 6-MP, converting it to 6-thiourea acid (6-TUA), 6-methylmercaptopurine (6-MMP), and precursors of the active 6-TGN, respectively (Figure 1).

The purine analog 6-TGN suppresses DNA replication by incorporating itself into nucleic acids. 6-TGN eventually inhibits T-lymphocyte proliferation, resulting in immunosuppression. AZA also suppresses numerous genes associated with intestinal inflammation and leukocyte trafficking to the gut, including tumor necrosis factor (TNF)-related apoptosis-inducing ligand, TNF receptor superfamily member 7, and alpha-4-integrin, inactivated T-lymphocytes, or by T-cell apoptosis induction by preventing CD28-dependent Rac1 protein stimulation [4, 5].

The discrepancy in patient responses to AZA may be due to genetic variations in *TPMT* and *NUDT15* [6]. The clinical response to AZA treatment is inversely associated with TPMT activity [7]. The high TPMT activity is linked to decreased 6-TGN and higher 6-MMP levels, which results in lower response rates and increased hepatotoxicity [8]. With careful monitoring for developing hepatotoxicity, the AZA dose can rapidly increase to therapeutic drug levels in individuals with high TPMT activity. In comparison, the low TPMT activity necessitates a lower initial dose and a more gradual dose elevation to avoid myelosuppression [8]. A genetic variation in the *TPMT* gene affects around 10% of the population, resulting in lower TPMT enzyme activity and greater 6-TGN levels, increasing the risk of potentially fatal myelosuppression after AZA therapy [9]. The *NUDT15 *gene encodes a nucleoside diphosphate-linked moiety X-type motif 15 that hydrolyzes the 6-TGN. This polymorphism causes azathioprine-induced myelosuppression in the *NUDT15* gene, which causes an accumulation of 6-TGN.

Efficacy

The effects of AZA for induction of remission in patients with UC are equal to placebo. Hence, it is not used to induce remission in this population [10,11]. A meta-analysis also revealed that AZA is ineffective for inducing remission in patients with active UC [12]. The Toronto UC Consensus Group does not recommend inducing remission solely with AZA [13]. Based on these findings and recommendations, AZA monotherapy should never be used to induce remission in patients with active UC. AZA has been combined with biologicals to induce remission for the past few years. Patients receiving infliximab and AZA in combination are more likely to achieve corticosteroid-free remission at 16 weeks in anti-TNF-naive moderate to severe UC than those receiving infliximab alone (40% vs. 22%) or AZA alone (24%) [14].

AZA is indicated for individuals who have failed or are intolerant to mesalamine therapy and require multiple doses of steroids to maintain remission [15]. The alternatives of colectomy or intensified medical therapy are frequently offered to patients with steroid-dependent UC who need more than two courses of steroids per year [16]. In 53% of patients treated with AZA, steroid-free endoscopic remission is achieved, compared with 21% of patients treated with mesalamine for steroid-dependent UC [17]. In patients with UC, co-prescribing mesalamine with AZA does not significantly benefit AZA alone in maintaining remission or steroid withdrawal [18, 19].

Ardizzone et al. reported that the cumulative annual steroid dose and relapse rates were significantly lower three years after initiating AZA therapy, and disease duration of three years resulted in more sustained steroid-free remission [17]. The early introduction of thiopurine treatment will result in more successful mucosal healing and enhanced long-term outcomes in patients. In patients with UC, the combination therapy of AZA and anti-TNF is more effective in maintaining remission and has a higher mucosal healing rate than AZA or biological monotherapy [14].

Dosage and prerequisites

Before initiating azathioprine therapy, the European Crohn's and Colitis Organization (ECCO) guidelines recommend a complete history of past infection, immunization, and environmental risk of infection, including tuberculosis; tuberculosis screening, and serology for Epstein-Barr virus (EBV), varicella-zoster virus (VZV), hepatitis B virus (HBV), hepatitis C virus (HCV), and human immunodeficiency virus (HIV) [20].

There are two types of dosing strategies for AZA; the first is empiric weight-based dosing, and the second is based on *TPMT* and *NUDT15* polymorphism testing. The dosing pattern for weight-based doses varies as well, with some clinicians preferring to start with a target dose of 2.0 to 2.5 mg/kg/day. In contrast, others begin with a low dose (1 mg/kg/day) and progressively increase the dose every 2 to 4 weeks to reach the target dose. AZA can be administered as a full dose, or dose escalation can be performed in the case of wild-type polymorphism of *TPMT/NUDT15*. Pretreatment TPMT activity assessment aids patients in achieving therapeutic response more quickly and affordably while reducing toxicity [21].

*TPMT* testing involves phenotyping (enzyme activity measurement) or genotype testing (single-nucleotide polymorphism analysis and mutation detection). *TPMT* genotyping is a polymorphism that can be wild type (89%), heterozygous (10%), or homozygous (0.5%). The data variability makes it difficult to determine which test should be explicitly used to diagnose heterozygous disorders [9,22]. More than 95% concordance rate between *TPMT* gene mutation and TPMT enzyme activity [7,9]. In forecasting the risk of leukopenia, enzyme activity assessment is more sensitive and cost-effective than genotyping [22]. Patients who have undergone a blood transfusion within the last three months should not be evaluated for enzyme activity. The *NUDT15 R139C* polymorphism is strongly linked to azathioprine-induced leukopenia [23]. The current approach shows that azathioprine should be started as a standard dose in wild-type patients and avoided in homozygous *TPMT/NUDT15* variations. Azathioprine should be initiated at 30-80% of the regular dose for heterozygous patients, with dose modifications every two to four weeks [24,25].

Patients with UC benefit clinically from the AZA therapy in maintaining the remission, but there is insufficient evidence about how long it should be taken. Hawthorne et al. suggest that maintenance therapy with AZA is effective for at least two years following remission in UC [26]. Fraser et al. found that the drug's efficacy is relatively well-maintained over five years in patients with UC [27].

Dose reduction or discontinuation may be considered in individuals at high risk of severe AZA-related adverse events with a low probability of relapse. Patients receiving just an immunomodulator have a recurrence rate of about 30% after stopping AZA for a year [28]. When combined with anti-TNF medication, termination of both therapies results in a recurrence rate of nearly 40% in one year [28]. Patients who received combination therapy with immunomodulators and infliximab (IFX) for at least six months had an IFX failure rate of 20% two years after immunomodulator termination, compared to the rate in patients who persisted with combination therapy [28]. Patients with severe UC show signs of active disease when thiopurines are stopped. Furthermore, when thiopurines are discontinued after a shorter treatment period, there is a higher relapse rate [29].

A multidisciplinary European expert panel recommends discontinuing thiopurine monotherapy after four years of clinical remission in CD patients. Furthermore, anti-TNF therapy can be suspended while continuing thiopurine therapy after two years of clinical remission in combination therapy [30]. However, no such agreement exists regarding the discontinuation of biological or immunomodulatory medication in patients with UC.

Dose optimization and drug interaction

Combining 5-ASA and thiopurine is commonly used to sustain remission in patients with UC. Due to TPMT suppression, 5-ASA enhances 6-TGN levels in 82-100% of patients [31-34]. The therapeutic efficacy of AZA is enhanced, and the risk of hepatotoxicity is reduced as a result of this therapeutic interaction. However, compared to thiopurine monotherapy (16%), this combination therapy raises the incidence of leucopenia by up to 47% [32,34,35]. When coupled with 5-ASA therapy, it is therefore recommended that the target dose of AZA be reduced by 25% [15,31,32,36].

Patients who are preferential 6-MMP metabolizers (14%) have greater 6-MMP levels and sub-therapeutic 6-TGN levels, resulting in poor therapeutic response and increased hepatotoxicity [37,38]. Adding xanthine oxidase inhibitors like allopurinol or febuxostat will increase therapeutic 6-TGN levels and reduce hepatotoxic 6-MMP metabolite levels in this subgroup of individuals [37-41]. Ansari et al. suggest that combining allopurinol with thiopurine therapy raises mean 6-TGN levels from 370 to 563 pmol/8 × 108 RBCs while lowering 6-MMP levels from 11,604 to 696 pmol/8 ×108 RBCs [41].

Though the inhibition of XO is thought to cause metabolite shifts to 6-TGN, other hypothesized theories revolve around the enzymatic pathways of AZA metabolism, such as TPMT inhibition (due to metabolite 6-thioxanthine) and increased hypoxanthine-guanine phosphoribosyltransferase (HPRT) activity, which makes more 6-MP available to the 6-TGN pathway [37,42-44]. Allopurinol plus low-dose AZA results in enhanced HPRT activity and, as a result, increased 6-TGN levels while decreasing 6-MMP/6-TGN ratios [42, 44]. It is recommended to take 100 mg of allopurinol daily to reduce AZA by at least 50%, with close monitoring for developing leukopenia [40,45].

Splitting the daily dose of AZA is another strategy for reducing side effects while maintaining therapeutic efficacy in preferential 6-MMP metabolizers. Shih et al. found that dividing the daily thiopurine dose reduces 6-MMP levels from 11785 to 5324 pmol/8 × 108 RBCs, resolving 6-MMP-associated adverse effects in 90% of patients without compromising clinical response or a significant decrease in 6-TGN levels [46].

Anti-TNF monotherapy (infliximab and adalimumab) effectively induces and maintains UC remission. Combining anti-TNF medication with thiopurines early during treatment can improve mucosal healing and reduce the need for surgery. Concurrent usage of thiopurine and anti-TNF decreases immunogenicity to biologics [47]. In the subset of patients with elevated 6-TGN levels, Roblin et al. observed a significant increase in 6-TGN concentration within one to three weeks following the first infliximab injection, as well as a better clinical response to infliximab [48]. Direct pharmacological interaction between AZA and biologics may be a possible explanation for this occurrence [47,49]. Infliximab has a well-established pharmacokinetic interaction compared to adalimumab [50]. The superior treatment response to anti-TNF and thiopurine combination therapy is attributed to immunogenicity suppression and enhanced 6-TGN levels, at least with infliximab.

Patients intolerant to AZA can be treated with 6-MP safely and effectively [51]. Despite having a history of AZA hypersensitivity, 60-75% tolerated 6-MP well, and tolerance was higher in patients with UC than in Crohn's disease (CD) patients [51,52]. Thioguanine (6-TG) is another treatment option for patients intolerant to AZA and 6-MP [53,54]. Bonaz et al. discovered that 46% of their thiopurine-intolerant patients were in clinical remission with 6-TG after six months and 79% after 12 months, with no severe side effects [54].

Monitoring

Regular hematologic monitoring is required to detect myelotoxicity, commonly manifested as leukopenia and, to a lesser extent, thrombocytopenia [55]. For delayed complications such as myelotoxicity, complete blood counts and liver function tests should be performed every week for the first month, every two weeks for the next two months, and then every three to four months [36]. Pretreatment genetic testing for *TPMT* and *NUDT15* does not prevent patients from being monitored, but it does aid in selecting the proper dose [56]. The amounts of 6-TGN and 6-MMP metabolites in erythrocytes can be used to monitor therapeutic medication levels and reduce the risk of toxicity. AZA metabolite monitoring should begin at least four weeks after starting or changing the drug. Because erythrocytes (RBC) lack the enzyme IMP that converts mercaptopurine to TGN, erythrocyte TGN is utilized as a "surrogate" pharmacokinetic measure for TGN in target cells: leukocytes or bone marrow [57]. A reversed-phase high-performance liquid chromatographic method developed by Lennard et al. should be used to quantify AZA intracellular metabolites in human RBCs [58]. When dose optimization is done based on 6-TGN levels, 80-90% of patients have a better clinical outcome [59]. However, most facilities presently monitor AZA metabolites for therapeutic failure or adverse effects.

The 6-TGN level is the only metabolite that correlates with the clinical response to AZA [60]. The therapeutic efficacy of AZA has been associated with 6-TGN levels > 235pmol/8 × 108RBCs, whereas the risk of leukopenia is associated with 6-TGN levels > 450pmol/8 × 108RBCs, and hepatotoxicity develops at 6-MMP levels > 5700 pmol/8 × 108RBCs [60]. Despite being on the same doses, patients in remission have greater average 6-TGN levels than those with active disease [61]. The relationship between 6-TGN levels and clinical remission rates was recently shown in a meta-analysis; the pooled odds ratio for clinical remission among patients with 6-TGN levels between 230 and 260 pmol/8 × 108RBCs was 3.15 [62].

Complications and adverse drug reactions

Due to major adverse medication events, 15-20% of patients with inflammatory bowel disease (IBD) discontinue thiopurine therapy after one month of treatment [7,63,64]. Adverse drug reactions can be dose-dependent, such as myelosuppression and hepatotoxicity, or dose-independent, such as pancreatitis and flu-like illness. The accumulation of 6-TGN causes myelosuppression, while 6-MMP levels are associated with hepatotoxicity. Low TPMT activity resulted in high 6-TGN levels and myelotoxicity, whereas high TPMT activity (>14 U/mL RBC) causes higher 6-MMP levels and hepatotoxicity [36]. Asians are more likely to have *NUDT15 R139C* gene variations, with reported myelosuppression rates of 3%, 20%, and 100% in the wild type, heterozygous, and homozygous populations, respectively [65]. Due to its high cost, the real-world image of genetic testing is currently limited. The most prevalent cause of myelosuppression with thiopurine is isolated leukopenia, which affects about 3% of patients per year of treatment [55]. Although myelosuppression can occur at any point during treatment, from 12 days to 27 years, it is more frequent during the first few months of treatment [55]. When the WBC falls below three ×109/l and 1×109/l, respectively, the dose of AZA should be dropped by 50% and stopped.

Because there are no universally accepted criteria for defining hepatotoxicity in thiopurine therapy, the rate of hepatotoxicity varies from one study to the next. In individuals with IBD, the rate of thiopurine-induced liver impairment is around 3% per patient-year [66]. The dose of thiopurines should be reduced by 50% if there is a significant increase in liver enzymes (no precise cut-off point) [66]. If the liver enzymes do not return to normal after reducing the thiopurine dose, therapy should be stopped. For patients who develop jaundice while receiving thiopurine treatment, these medications should be discontinued completely without tapering [66]. Nausea, vomiting, and abdominal discomfort are common and dose-dependent gastrointestinal side effects. Taking the drug at bedtime or half the daily dose aids in the relief of symptoms [46]. Switching from AZA to 6-MP also alleviates symptoms, as these symptoms are associated with the imidazole derivative produced when AZA is transformed into 6-MP [51]. Pancreatitis typically occurs in 4-7% of thiopurine-treated patients and most of them have mild to moderate-severe pancreatitis [64,67]. The reintroduction of thiopurines after a pancreatitis episode is contraindicated because the mechanism is an idiosyncratic reaction linked to the *class II HLA* region [68]. Gallego-Gutierrez et al. found that 6-MP was effective in two pediatric cases of AZA-induced pancreatitis [69]. In patients receiving thiopurine therapy, infection susceptibility is a major concern. The incidence of infections in patients using thiopurines has been about 2%, with viral infections such as cytomegalovirus, EBV, VZV, and herpes simplex virus being the most prevalent [64].

Long-term (>2 years) use of thiopurines as monotherapy or in combination with anti-TNF has been linked to the development of lymphoma [70-72], urinary tract cancer [73], and non-melanoma skin malignancies [74,75]. The data on the link between lymphoma and thiopurine therapy in population-based research has been inconsistent [70,71,76-78]. A meta-analysis found that patients treated with immunomodulators had a relative risk of a 4.18 higher incidence of lymphoma [72]. In patients with IBD, thiopurine therapy and disease severity are linked to the development of lymphoma [72]. It has been observed that there is a clear link between EBV-associated lymphoma and thiopurine use [79]. Overall, the benefits of thiopurine administration outweigh the risk of lymphoma [79].

Special situations

*Vaccination* 

Patients who get AZA are considered immuno-compromised, and live vaccinations are not recommended. Vaccination with live vaccines should be done at least one month before starting AZA and three months after stopping it. Peptides and polysaccharide vaccinations are safe during AZA therapy. However, the response to polysaccharide vaccines may be impaired as they trigger a poor memory T-cells response [20].

Surgery

The use of AZA before surgery for UC-related purposes such as colectomy does not raise the risk of complications [80,81]. A recent meta-analysis found that using immunomodulators during both elective and emergency surgery in UC does not enhance the incidence of wound-related complications, intra-abdominal, or extra-abdominal infections [82].

Lactation and Pregnancy

AZA can cross the placental barrier. However, 6-TGN levels in the mother are lower, and 6-MMP levels in fetal RBCs are not detectable [83]. Multiple meta-analyses have revealed that AZA does not increase the risk of low birth weight or congenital abnormalities and can be safely taken throughout pregnancy [84-86]. There is mixed evidence regarding preterm delivery, which is linked to UC activity rather than AZA treatment. It is not suggested to initiate AZA during pregnancy due to the slower therapeutic effect and the unpredictability of complications. As anti-TNF and AZA-treated infants are more vulnerable to infections, the preferred therapeutic strategy is to discontinue AZA while continuing anti-TNF. AZA has been detected in breast milk in relatively small amounts and is considered "probably safe" during lactation [86]. A significant amount of AZA is released into breastmilk within four hours following drug administration; therefore, breastfeeding reduces drug exposure to newborns after these hours.

Children

Pediatric-onset UC has a higher incidence of extensive colitis and aggressive disease course. The prevalence of steroid-dependent UC is higher, and immunomodulators should be started as soon as possible [87]. The disadvantage of early immunomodulator use is that it leads to many years of drug exposure and risks the development of a rare type of lymphoma called hepatosplenic T-cell lymphoma (HSTCL). Azathioprine should be used with caution in children, with frequent monitoring and a close eye on the development of malignancy [87].

Coronavirus Disease 2019 (COVID-19)

In UC patients, the likelihood of COVID-19 infection and the severity of the disease did not rise [88]. There is no indication that AZA use raises the risk of COVID-19 infection. AZA is not recommended to be discontinued in patients with UC; however, close monitoring is required in these individuals, especially if anti-TNF is also administered [89].

## Conclusions

Azathioprine is an efficient and cost-effective medicine for UC patients who want to stay in remission for a long time. AZA therapy necessitates a thorough pre-therapy clinical and laboratory evaluation and genetic analysis. Both patients and physicians should be aware of potential consequences, such as cancer, and should be tested regularly. An effective therapeutic strategy should be developed to reduce potential problems while retaining the therapeutic efficacy of AZA therapy. Due to the availability of biologics and small molecules, it is more important than ever to make a customized treatment plan while managing patients with UC, especially in resource-constrained settings.
